# A pilot study on the effects of a team building process on the perception of work environment in an integrative hospital for neurological rehabilitation

**DOI:** 10.1186/1472-6882-10-10

**Published:** 2010-03-09

**Authors:** Thomas Ostermann, Mathias Bertram, Arndt Büssing

**Affiliations:** 1 Centre of Integrative Medicine, University of Witten/Herdecke, Gerhard-Kienle-Weg 4, 58313 Herdecke, Germany; 2 Institute of Nursing Science, University of Witten/Herdecke, Stockumer Str. 10, 58448 Witten, Germany

## Abstract

**Background:**

Neurological rehabilitation is one of the most care-intensive challenges in the health care system requiring specialist therapeutic and nursing knowledge. In this descriptive pilot study, we investigated the effects of a team building process on perceived work environment, self-ascribed professional competence, life satisfaction, and client satisfaction in an anthroposophic specialized hospital for neurological rehabilitation. The team-building process consisted of didactic instruction and training in problem-solving, teambuilding and constructive conflict resolution.

**Methods:**

Seventy seven staff members and 44 patients' relatives were asked to complete a survey that included the Work Environment Scale (WES-10), a Life Satisfaction Scale (BMLSS), the Conviction of Therapeutic Competency (CTC) scale and the Client Satisfaction Questionnaire (CSQ-8). To evaluate the outcome of the team building process, we analyzed changes over time in the WES-10 subscales. Additionally the interrelationship between the WES-10 subscales with other subscales and with sociodemographic parameters like age, gender was calculated by means of a bivariate correlation analysis.

**Results:**

The team building process had a significant positive effect on perceived work environment in only one area. There was a significant improvement in the ward staffs' perception of their ability to constructively resolve conflicts 3 years after inception of the team building process than there was before inception. However, even in a unit that utilized holistic treatment and nursing in the care of severely disable patients, such care necessitating a very heavy workload, the measurements on the Self Realization, Life Satisfaction and Conviction of Therapeutic Competency scales remained high and unchanged over the three year time period of the study.

**Conclusions:**

Strategic interventions might be an option to improve interpersonal relationships and finally quality of patient care.

## Background

The nature of the environment in which a medical staff does their work plays an important role in job satisfaction and performance. Several studies already investigated the relationship between the milieu in which health professionals work and the impact on job satisfaction. They proved that poor work environment is associated with reduced job satisfaction, absenteeism, somatic complaints, burnout and depression [1-3]. Moreover, poor work environment might also influence the work performance negatively, and might also promote negative and cynical attitudes towards patients and colleagues [4], which in turns will have an impact on the patients' satisfaction and their relatives' satisfaction. Finally there is evidence that work milieu is probably one of the main reasons for the high staff turnover rate and poor inpatient satisfaction and outcome [5,6].

Neurological rehabilitation today is one of the most care-intensive challenges in the health care system [7]. It goes beyond the rather narrow borders of conventional physical rehabilitation as it deals with the psychological and biographical consequences of the disease for the patients as well as for their relatives. Thus, neurological rehabilitation is a very individual form of rehabilitation requiring specialist therapeutic and nursing knowledge and training to be sequentially adapted to the single patient. There is no doubt that this task has to be taken by a comprehensive multidisciplinary rehabilitation team consisting of physicians, therapists and nurses [8]. This on the one hand leads to a high workload for the complete team and on the other hand might cause problems with respect to the self-image of the involved persons and their loyalty towards their own profession. Thus, team building measures to decrease conflicts and to enhance satisfaction and identification of the team with their work are seen as essential parts of modern clinical quality management. Organizational research has demonstrated that team building can enhance management of complex client problems with improved staff morale, client satisfaction and perceived work environment [9].

Particularly work environment is a unique feature in hospitals with an integrative approach on patient treatment. According to Melchart et al. [10], structural features like facility characteristics, medical equipment, constructional features or internal communication play an important role in the quality profile of integrative rehabilitation clinics. Thus, the above mentioned aspects may play a more central role in neurological or other critical care units with an integrative medical care program than conventional medical care programs.

In this descriptive pilot study, we therefore intended to investigate the effects of a team building process on perceived work environment, self-ascribed professional competence, life satisfaction, and client satisfaction in an anthroposophical oriented specialized hospital for neurological rehabilitation for severe neurological diseases and acquired brain injury in a three-year course of time.

## Methods

### Setting

The Raphael Medical Centre (RMC) is a registered hospital for rehabilitation of patients with neurological diseases and acquired brain injury offering both short-term and long term placement. This hospital promotes the integrative approach to rehabilitation and healing based on Anthroposophic Medicine in conjunction with conventional therapies. The specific therapy consists of various forms of Physiotherapy and Hydrotherapy, Speech Therapy, Music and Art Therapy, Occupational Therapy, Chiropractic and Psychological Care.

Additionally the following unique anthroposophic treatments are applied

• **Neurofunctional Reorganisation according to Padovan**: Neurofunctional Reorganisation is based on men's natural development phases (i.e., rolling, creeping, crawling). These phases according to Padovan are significantly important for the maturation of the central nervous system and going through all these phases again stimulates the nervous system to reorganise [11].

• **Eurythmy Therapy**: Introduced by Rudolf Steiner in 1911, Eurythmy Therapy can be described as an active exercise therapy, involving cognitive, emotional and volitional elements in which speech movements are transposed into exercises which address the patient's capability to soul expression and strengthen his salutogenetic resources [12].

• **Oil dispersion baths**: In 1920 Rudolf Steiner suggested the use of finely-dispersed oil in water in. If a patient's 'warmth organism' is affected or weakened, one may, according to his theory, connect it to the 'warmth processes' of nature, i.e., the 'oil forming process' of flowering plants, and thus introduce finely dispersed essential oil into a bath to strengthen the weakened 'warmth organism' [13].

• **Rhythmical Massage/Embrocation**: Rhythmic massage/embrocation invented by Ita Wegman in the 1920^th ^is based on a standardized series of manual movements and directional strokes and involves the application of a lubricant (oil, emulsion or ointment) to a specific area of the body by sliding motions of the hands with the intensity of touch alternating rhythmically between the two hands. Directional stroking movements are oriented to respective organ areas and local muscle contours [14].

### Purpose of the study

The goal of the study was to evaluate the effects of a team building process on perceived work environment and client satisfaction. Additionally we analysed the relationship of self-ascribed professional competence, life satisfaction and sociodemographic parameters with the outcome of the team building process.

### Study design

To assess the effects of a team-building process on staff's perception of the work environment, we chose a time-series design with three measurement points:

T0: January 2007 (Baseline), before the team building process started

T1: January 2008 (Interim), during the team building process and

T2: January 2009 (Post), after team building process was formally finished

The team building process included the development of skills for constructive conflict resolution, learning to be aware of factors that influence team performance, actions to improve team-effectiveness and the planning of goals, decisions-making, resolving of differences, and problem solving.

The study was based on questionnaire data from physicians, therapists and nurses on the one hand and patients' relatives on the other. Neither the head of the hospital nor the evaluators were able to identify the staff members. All questionnaires were strictly anonymized and externally stored at the University of Witten/Herdecke (Germany). Thus, we applied the specific conditions and requirements for "Good Practice of Secondary Data Analysis" provided in [15] to this study and the study was vetted by the hospitals responsible data security and protection official. All staff members were informed of the purpose of the study and were assured of confidentiality, and gave informed consent to participate. Due to the small size of the team and to assure confidentially, we renounced on acquiring any basic data like age, gender, and profession along with the questionnaires at T0 and T1. Only at the end of the evaluation phase (T2), we extended our questions towards basic data.

To obtain an additional perspective on the situation at the hospital, we also asked relatives about their satisfaction with therapy and services at the hospital.

### Research instruments

The following measures were used in the survey:

#### Work Environment Scale

This 10 item-scale was originally developed with mental health workers to measure the impact of the work environment on job satisfaction [16]. The *Work Environment Scale (WES-10) *contains the following subscales:

• Self-realization (SR) - the extent to which workers feel supported in the work environment, have confidence, and can use their knowledge on the job (Items 1,2,5,6). Cronbach's alpha: 0.85.

• Workload (WL) - the number of tasks assigned to employees and the degree to which they believe they must be in "two places at once" (Items 9 & 10). Cronbach's alpha = 0.84

• Conflict (C) - the degree to which employees have interpersonal conflicts and loyalty problems on the job (Items 7 & 8)., Cronbach's alpha = 0.69.

• Nervousness (N) - the extent of worry, nervousness, and tension workers feel on the job (Items 3 & 4). Cronbach's alpha = 0.66.

Scores for each scale range from 1 to 5, with higher scores indicated a higher level of that aspect of work. The WES-10 has been validated on staff in a public mental health facility with satisfactory internal consistency reliability values given above.

#### Conviction of Therapeutic Competency

Self ascribed professional competence was measured with the 5-item scale *Conviction of Therapeutic competency *(CTC) from the the ThSER questionnaire (acronym of "Therapeutic Self Efficacy and Relationship") [17] including the following items: Patients can fully rely on my professional skills; Patients can always rely on my professional advise; I always do know which treatment/care is the best for my patient; I do know that I can trust my own skills and thus I can face even difficult medical problems with serenity; Whatever may come across in patient care, I will get on with it. The items are scored on a on a 5-point scale from disagreement to agreement (0 - does not apply at all; 1 - does not truly apply; 2 - don't know; 3 - applies quite a bit; 4 - applies very much). The sum score was referred to a 100% level (4 "applied very much" = 100%). A validation of this scale in German physicians found a high internal reliability (Cronbach's alpha = 0.81).

#### Brief multidimensional Life Satisfaction Scale

The eight items of the *Brief Multidimensional Life satisfaction scale *(BMLSS) [18] refer to intrinsic dimensions (Myself, Overall Life), social dimensions (Friendships, Family life), external dimension (Work, Where I live), and the perspective dimension (Financial Situation, Future Prospects) of subjective life satisfaction. The items are scored on a 7-point scale from dissatisfaction to satisfaction (0 - Terrible; 1 - Unhappy; 2 - Mostly dissatisfied; 3 - Mixed (about equally satisfied and dissatisfied); 4 - Mostly satisfied; 5 - Pleased; 6 - Delighted). The BMLSS sum score was referred to a 100% level ("Delighted"). Previous analyses have shown that the BMLSS correlated negatively with Depression and Anxiety, Fatigue, and positively with SF-12's mental health and to a weaker content also with physical health [18]. Internal reliability was 0.87 (Cronbach's alpha).

#### Client Satisfaction Questionnaire

The *Client Satisfaction Questionnaire *(CSQ-8) is an 8-item, easily scored and administered measurement developed by Larsen et al. [19] which was designed to measure client satisfaction with services. The items of the CSQ-8 were selected on the basis of ratings by mental health professionals of a number of items that could be related to client satisfaction and by subsequent factor analysis. The CSQ-8 is uni-dimensional, yielding a homogeneous estimate of general satisfaction with services. The CSQ-8 has been extensively studied, and while it is not necessarily a measure of a client's perceptions of gain from treatment or outcome, it does elicit the client's perspective on the value of services received. The CSQ-8 is easily scored by summing the individual item scores to produce a range of 8 to 32, with high scores indicating greater satisfaction. Depending on the population, the internal consistency of the CSQ-8 can be regarded as being high with values of Cronbach's alphas between 0.83 and 0.96.

To assess special areas of satisfaction, 18 further items were administered to the relatives. These items asked for their therapeutic satisfaction (12 items) and general satisfactory with the structural qualites of the RMC (6 items). Both CSQ-8 and the additional satisfaction questionnaire were administered to the relatives in the time between T_0 _and T_1_.

### Statistics

Although the original scales given by Rossberg et al. [16] were used for our evaluation, we also performed a factor analysis on the WES-10 using Principal Component Analysis and Varimax Rotation to detect principle differences to our sample.

Further statistical analysis included descriptive statistics and the calculation of subscale means with 95% confidence intervals. Additionally the interrelationship between the WES-10 subscales with other subscales and with socio-demographic parameters like age, gender was calculated by means of a bivariat correlation analysis and nonparametric tests (Kruskal-Wallis test and Mann-Whitney U-test).

To evaluate the outcome of the team building process, we analysed the changes in the WES-10 subscales and the single items by means of Mann-Whitney U-Test and Chi-Square-Test statistics.

## Results

### Staff characteristics

The staff consisted of 55 nurses and healthcare workers, 19 therapists and 3 physicians (female: 65.3%) with a mean age of 40.0 ± 13.2 years. These professions were caring for 44 patients. Forty members of the staff (51.9%) completed the questionnaires at baseline (T_0_), 50 (64.9%) within the process of team building (T_1_) and 47 (61.0%) after the team building process (T_2_). From 44 questionnaires send to the relatives, a total of 18 (40.9%) responded with a completed questionnaire.

Life Satisfaction was quite high among the staff members (76.0 ± 14.0), and did not significantly differ between the professions (F = 1.5; p = 0.226). There were no gender specific effects.

Conviction of Therapeutic Competency was high among the staff members (74.7 ± 21.8), and was in trend higher in therapists and nurses than in the other professions (F = 2.7; p = 0.074). Although the score of women were somewhat lower than in male persons, the differences were not significant (F = 0.7; p = 0.4)

### Factor structure and external validity of the WES-10

Table 1 shows the mean values of each item of the WES-10 questionnaire, and results of a new factor analysis which was performed to assure quality of the instrument in this unique setting. Principal Component Analysis with Varimax Rotation and Kaiser-Normalization converged in 7 iterations into a 4-factor solution which explained 67.5% of the total variance. Factors II and III of this new analysis had an identical item grouping with the original scales "Workload" (Items 9 and 10) and "Conflict" of Rossberg *et al. *(Items 7 and 8). The other items were grouped differently, indicating a closer connection of "Nervousness" and "Self-Realization" in this survey, which was also approved by a moderate inverse correlation (r = -0.42) between these scales in Table 2. Non of the work environment scales correlated with Conviction of Therapeutic Competency, and with respect to life satisfaction just "Nervousness" correlated inversely (r = -0.4).

**Table 1:** Distribution of responses and item mean values of each item on the WES-10 and results of the factor analysis (items marked with * are recoded for the calculation of the subscales)

Item No.	Item	Original Scale^§^	Resulting factor loadings				Item Mean (SD) [Scores 1-5]		
			
			I	II	III	IV	T_0_	T_1_	T_2_
1	Does what you do on the ward give you a chance to see how good your abilities really are?	SR	0.701				3.4 (1.0)	3.4 (0.8)	3.4 (0.8)
2	Does what you do on the ward help you to have more confidence in yourself?	SR	0.780				3.4 (0.9)	3.4 (0.9)	3.4 (0.8)
3	To what extent do you feel nervous or tense on this ward?	N		0.715			2.4 (1.1)	2.3 (1.0)	2.1 (0.9)
4	How often does it happen that you are worried about going to work?*	N		0.817			2.3 (1.1)	2.3 (1.2)	2.3 (1.0)
5	To what extent do you feel that you get the support you need, when you are faced with difficult treatment problems?*	SR		0.568	0.436		3.3 (1.1)	3.5 (1.1)	3.4 (1.1)
6	To what extent do you find that you can use yourself, your knowledge and experience in the work here on this ward?	SR	0.820				3.2 (1.0)	3.6 (0.8)	3.4 (0.8)
7	To what extent do you find that the patient treatment is complicated by conflicts among the staff members?	C			0.824		2.9 (1.1)	2.5 (1.0)	2.4 (1.2)
8	To what extent do you find that it can be difficult to reconcile loyalty towards your team with loyalty towards your own profession?	C			0.750		2.8 (0.9)	2.4 (1.0)	2.3 (1.1)
9	What do you think about the number of tasks imposed on you?	WL		0.341		0.725	3.4 (0.7)	3.4 (0.9)	3.6 (0.7)
10	How often does it happen that you have a feeling that you should have been on several places at the same time?*	WL				0.803	3.4 (1.1)	3.5 (1.0)	3.2 (1.1)

^§ ^Rossberg et al., 2004 [15]

**Table 2:** Mean values and Interrelationship between the WES-10 subscales given by Spearmans's correlation coefficients

Scale values of the RMC Mean (SD)				Scale values of psychiatric wards^§^	Inter-Correlation of the scales				Correlation with other scales	
						
Scale	*T*_0_	*T*_1_	*T*_2_	Mean (SD)	*SR*	*WL*	*C*	*N*	*BMLSS*	*CTC*
***SR***	3.31 (0.73)	3.45 (0.68)	3.41 (0.61)	3.73 (0.23)	X	-0.06	0.34**	-0.42**	0.21	0.10
**WL**	3.40 (0.77)	3.45 (0.81)	3.40 (0.76)	3.41 (0.36)		X	0.23*	0.28*	-0.13	0.02
**C**	2.84 (0.86)	2.42* (0.87)	2.36* (0.97)	2.06 (0.28)			X	0.31**	-0.18	0.08
**N**	2.34 (1.00)	2.29 (0.99)	2.28 (0.90)	1.98 (0.21)				X	-0.43**	-0.09

Abbreviations: WES-SF - Self-realization component of the Work Environment Scale; WES-WL - Workload; WES-C - Conflict; WES-N - Nervousness; BMLSS - Life Satisfaction Sum Score; CTC - Conviction of Therapeutic Competency, Client Satisfaction Questionnaire (CSQ-8)

** p < 0.01; * p < 0.05

^§ ^Rossberg et al., 2004 [15]

Item Means and Compliance of the staff were quite low in items 3 and 4 (Nervousness of the primary scale) and 7 and 8 (Conflict). With 11 non-respondents at T_0_, items 7 and 8 of the "Conflict" scale did not only show the highest rate of non-respondents (27.5%) among the staff, but also offer quite high values of dissatisfaction resulting in a high "Conflict" score of 2.84 compared to the reference value of 2.06 from psychiatric wards (Table 3).

**Table 3:** Distribution of responses on the items of the "Conflict"-subscale on the WES-10 before, within and after the team building process

		To what extent do you find that the patient treatment is complicated by conflicts among the staff members?					
**Timepoint**		**not at all**	**to a small extend**	**to some extend**	**to a large extend**	**to a very large extend**	**Total**

**T_0 _(2007): Baseline**	N (%)	2 (6.9%)	8 (27.6%)	12 (41.4%)	4 (13.8%)	3 (10.3%)	29
**T_1 _(2008): Interim**	N (%)	6 (12.8%)	20 (42.6%)	14 (29.8%)	6 (12.8%)	1 (2.1%)	47
**T_2 _(2009): Post**	N (%)	13 (30.2%)	10 (23.3%)	13 (30.2%)	4 (9.3%)	3 (7.0%)	43

		**To what extent do you find that it can be difficult to reconcile loyalty towards your team with loyalty towards your own profession?**					

**T_0 _(2007): Baseline**	N (%)	3 (10.3%)	6 (20.7%)	16 (55.2%)	3 (10.3%)	1 (3.4%)	29

**T_1 _(2008): Interim**	N (%)	11 (22.9%)	15 (31.3%)	15 (31.3%)	6 (12.5%)	1 (2.1%)	47

**T_2 _(2009): Post**	N (%)	13 (28.3%)	14 (30.4%)	13 (28.3%)	5 (10.9%)	1 (2.2%)	43

### Outcome of the team building process

Within the process of team building, the conflict scale significantly decresed and in parallel compliance normalized as well. A closer analysis of the underlying item revealed that the change in "Conflict" was equally due to the change in both underlying items (Table 3). While at baseline (T_0_) almost 25% of the staff agreed that patient treatment was complicated by conflicts among the staff members (Item 7), the agreement decreased within the process of team building (T_1_) to 15% which was almost the same level after the team building process at T_2 _(16%) (p = 0.12, Chi-Square-Test). The same development was seen in item 8: At baseline (T_0_) 55% to some extent found it difficult to reconcile loyalty towards the team with loyalty towards their profession and only 31% did not have or only in a small extend had loyalty problems the interim percentages at T_1 _were reversed: 54% reported to have no or minor loyalty problems and only 31% to some extent found it difficult to reconcile loyalty which again improved to values of 58% and 28% respectively (Table 3). This development turned out to be statistically significant (p = 0.03, Chi-Square-test).

The scores of the "Workload" scale remained more or less unchanged throughout the whole process, and were similar like the reference values of psychiatric wards (Rossberg et al., 2004). In contrast scale-means of "Nervousness" slighly decreased within the process of team building, but still tended to be about 15% lower than those found in the staff of psychiatric wards. The "Self-Realization" score slighly improved during time, and was about 10% lower than the reference value (Table 2).

### Client Satisfaction

**Figure 1:**
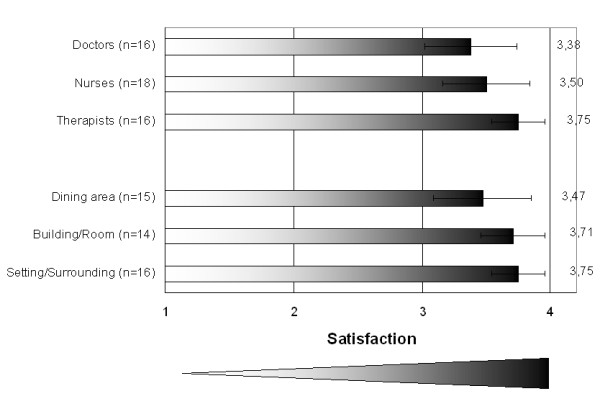
**Satisfaction of patient's relatives with structural qualities (Mean and 95% Confidence Interval) from 1 = very unsatisfied to 4 = very satisfied sorted by level of satisfaction**.

The appreciation of the staff's work by the relatives is an indirect measure of the quality of their work and the hospitality of the rehabilitation unit, and thus we measured the clients' satisfaction, too. Table 4 shows the distribution of responses to each item on the Client Satisfaction Questionnaire given to the relatives of the patients. With a total score on the CSQ-8 of 28.4 (SD = 2.95) out of a maximum of 32, the survey of relatives' satisfaction indicated that most of the relatives were very satisfied with the hospital service. They rated their overall satisfaction as strongly positive (55.6%) or at least moderately positive (44.6%). Nevertheless, in this setting one may expect positive answers of the relatives. Thus, it is worth to mention, that two topics, i.e. "Service desired" and "Program met needs", reached only moderate positive results (66.7% respectively 61.1%). On the other hand, 72.2% would return for counselling if they needed it in the future and would recommend the hospital to a friend in a similar situation. This goes alongside with the results of the additional satisfactory questions (Fig 1).

**Table 4:** Client satisfaction - Distribution of responses (%) and item mean values of each item on the CSQ-8 (N = 18)

Item	Strongly Negative %	Moderately Negative %	Moderately Positive %	Strongly Positive %	Missing %	Item Mean (SD)
1. Quality of Service	0	0	44.4	55.6	0	3.56 (0.51)
2. Service desired	0	0	66.7	27.8	5.5	3.29 (0.47)
3. Program met needs	0	0	61.1	38.9	0	3.39 (0.50)
4. Would recommend program to friend	0	0	27.8	72.2	0	3.72 (0.46)
5. Amount of help received	0	0	38.9	61.1	0	3.61 (0.50)
6. Services helped with problems	0	0	44.4	55.6	0	3.56 (0.51)
7. Overall satisfaction	0	0	44.4	55.6	0	3.56 (0.51)
8. Would return if in need	0	0	27.8	72.2	0	3.72 (0.46)

				*Total score*		**28.41 (2,95)**

Three of four relatives (78%) were very satisfied with building and rooms, 67% reported a very high satisfaction with the setting and surrounding and every second relative was very satisfied with the dining area (50%). Most of the relatives were also very satisfied with the care givers at the RMC (66% with nurses, 67% with therapists, 44% with doctors). Despite the high satisfaction with the nursing care, a major obstacle reported additionally by some of the relatives was given by the language barrier between them and nurses/therapists whose mother tongue is not English.

**Figure 2:**
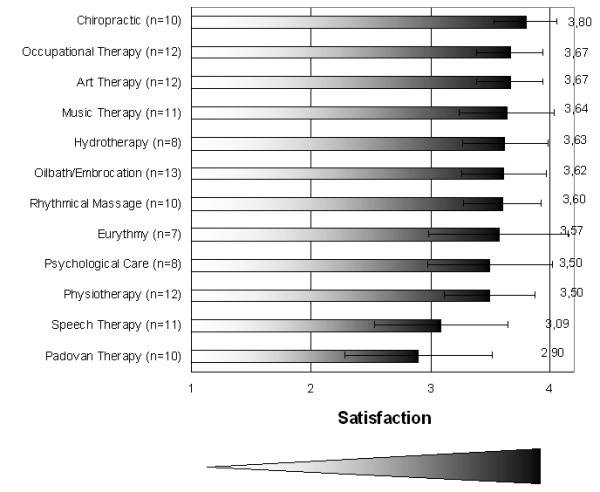
**Satisfaction of patient's relatives with therapies (Mean and 95% Confidence Interval) from 1 = very unsatisfied to 4 = very satisfied sorted by level of satisfaction**.

The high satisfaction with the therapists in general is also reflected in the therapies (Fig. 2). Although the responder rate for these questions ranged between 39% (Eurythmy) and 72% (Oil bath/Embrocation), the mean satisfactory values ranged between 2.9 and 3.8, indicating a high level of satisfaction with the therapies. However, Speech Therapy and Padovan Therapy reached the lowest level of satisfaction among the relatives. A possible explanation could be a poorer understanding of the therapeutic background of these unique treatments, or that they did not expect strong positive effect at all.

## Discussion

Although research on integrative approaches has increased over the last decades most of the research has been done outside critical care environments and thus the use of complementary therapies in critical care settings is watched carefully. However, according to a review of Sparber [20], critical care nurses have been leaders in this field and have successfully integrated complementary therapies in their work environment. Our article for the first time reports on the work environment and satisfaction of staff and patients' relatives with an integrative hospital for rehabilitation of severely neurological disabled patients.

Compared to the data from the psychiatric wards, we found a higher potential of conflict in the staff while, with respect to the small amount of participants, all other values were comparable with the reference values. Also in contrast to the original validation, we found a moderate negative correlation between Self Realization and Nervousness, which in our opinion is quite consistent: high values in Self Realization should go alongside with low values of Nervousness within the staff. In line with this, we found a moderate negative correlation between Nervousness and Life Satisfaction, while none of the other scales correlated with this dimension. However, none of the work environment scales correlated with Conviction of Therapeutic Competency, indicating that particularly professional competency is a dimension independent from positive or negative aspects of work environment.

Another important finding is that the subjective feeling of workload in our study remained stable within the three years and in its magnitude of 3.4 points was similar to reference values of psychiatric wards. This result might contribute to eradicate the prejudice that integrative approaches in day to day health care are related to a higher burden of workload for the halth care givers.

## Limitations

The study has some limitations which have to be taken into account when discussing the results. First, the small sample size and the low response rates limit the use of multivariate statistics and thus do not allow to generalize our results as it would have been preferable. Secondly, we also did not have any additional data on the staff to draw a more differentiated picture, i.e., which staff group improved the most from the team building process. Thirdly, due to organisational and compliance aspects we did not have the opportunity to collect longitudinal data on self-ascribed competency, life satisfaction and client satisfaction which would have made the results of the study more comprehensive. Finally we cannot exclude potential observation bias associated with the setting, i.e., the Hawthorne effect: staff members might have changed their behaviour, simply because they were evaluated with a questionnaire survey. Finally the survey population in this special case of an integrative hospital might not be seen as representative for other clinical settings in neurological care.

Despite of these limitations, one may nevertheless draw some general conclusion from this study. Although there have only been a few studies on the impact of multidisciplinary team building processes on patient care, our analysis is in line with studies demonstrating the importance of such interventions for interpersonal relationships and satisfaction with work environment: In accordance with Adams & Bond [21], and Sheward et al. [22], we found that the development of cohesive working relationships measured implicitly by the WES-conflict-scale is the most important factor, while perceptions of the workload and self-realization are of less importance. Particularly with respect to multicultural team building, our results underpin the results of Hope et al. [23], who found that team-building measures resulted in an improved interdisciplinary understanding, team atmosphere, and teamwork skills. Similarly, the study of Amos et al. [24] found that the introduction of team-building activities resulted in improved interpersonal relationships and a higher job satisfaction.

Another study of DiMeglio et al. [25] examined a team-building approach in registered nurses in an acute care setting and likewise found improvement in group cohesion and nurse satisfaction.

Our results suggest that the team building process in this respective unit has to be further strengthened in the future. There is still a continual need to work with staff in the areas of listening, feedback, and conflict management. Particularly in the given case of a holistic approach to neurological rehabilitation of severely disabled patients, transparency in goal planning strategies as well as team building measures and shared decision making are still important tasks [26]. Working continuously on these issues will not only support the working atmosphere but may also help to avoid therapeutic overload and misbalance for the patients. Moreover, to enhance the efficacy of a coordinated multidisciplinary rehabilitation approach special measures like supervision in the long rung might help to decrease unspoken conflicts within the team.

Although we found some potential for conflicts within the team structure, this did not affect the overall satisfaction of the relatives with the care delivered, indicating social competence in terms of client satisfaction. Moreover, there was a high appreciation of the staff's work by the relatives, which in turns is a strong motivation and enhances their overall satisfaction.

However, clinical experience has shown that relatives of patients in vegetative state are usually grateful for attempts like neuro-developmental interventions, which alter the patient's state and accordingly most relatives are found to answer to be satisfied with all aspects of care although the improvement of the patient is only marginal [27]. Thus, the relation between relative's satisfaction and quality of care has to be further explored as some dimensions of care are of relevance for relatives' satisfaction, particularly when the patients itself are not able to communicate. Questions on the satisfaction with the service desired (CSQ-2) or if the program met actual needs of the patients (CSQ-3) linked into this direction and emphasize the importance of an essential concept of integrative care.

## Conclusions

Even in a rehabilitation unit caring for severely disabled patients with an emphasis on holistic treatment and nursing and care, the work environment is comparable with psychiatric wards. Although the work load was high, both the Self Realisation but also Life Satisfaction and Conviction of Therapeutic Competency were high. Particularly Conflict, which correlated with Nervousness, improved within the time course, indicating that strategic interventions might be an option to improve interpersonal relationships and finally quality of patient care. Not only in this case, there is an obvious need for regular surveys on the medical staffs' perception of their work environment [28] and relatives' satisfaction to measure the quality of health service provided. The tools CSQ-8 and WES-10 have been proven to be reliable performance indicators for such evaluations. Our approach might also be useful for evaluating future changes and developments as suggested by Blau et al. [29], not only for the RMC but for all care-intensive wards, and to monitor and improve the clinical working environment.

## Competing interests

TO and AB received financial support with a grant of the Raphael Medical Centre, Hildenborough, Kent, Great Britain. The authors do not have any competing interests.

## Authors' contributions

TO designed the study, performed statistical analysis, and drafted the manuscript. AB and MB contributed to draft the manuscript. All authors read and approved the final manuscript.

## Pre-publication history

The pre-publication history for this paper can be accessed here:

http://www.biomedcentral.com/1472-6882/10/10/prepub
